# Modeling rapidly disseminating infectious disease during mass gatherings

**DOI:** 10.1186/1741-7015-10-159

**Published:** 2012-12-07

**Authors:** Gerardo Chowell, Hiroshi Nishiura, Cécile Viboud

**Affiliations:** 1 School of Human Evolution and Social Change, Arizona State University, 900 S. Cady Mall, Tempe, 85287-2402, Arizona, USA; 2 Division of Epidemiology and Population Studies, Fogarty International Center, National Institutes of Health, 31 Center Dr, MSC 2220, Bethesda, 20892-2220, Maryland, USA; 3 School of Public Health, The University of Hong Kong, Cyberport Road 100, Pokfulam, Hong Kong Special Administrative Region, China; 4 PRESTO (Precursory Research for Embryonic Science and Technology), Japan Science and Technology Agency, Honcho Kawaguchi 4-1-8, Saitama 332-0012, Japan

**Keywords:** Model, mathematical, epidemic, outbreaks, epidemiology, mass gathering, school closure, clustering, reactive vaccination, movement, social networks/

## Abstract

We discuss models for rapidly disseminating infectious diseases during mass gatherings (MGs), using influenza as a case study. Recent innovations in modeling and forecasting influenza transmission dynamics at local, regional, and global scales have made influenza a particularly attractive model scenario for MG. We discuss the behavioral, medical, and population factors for modeling MG disease transmission, review existing model formulations, and highlight key data and modeling gaps related to modeling MG disease transmission. We argue that the proposed improvements will help integrate infectious-disease models in MG health contingency plans in the near future, echoing modeling efforts that have helped shape influenza pandemic preparedness plans in recent years.

## Background

Mass gatherings (MGs) occur around the world on a relatively frequent basis, and include events as diverse as sport, religious, and educational activities [1]. MGs are typically defined as the influx of a large number of people at a specific location, for a specific purpose, and for a defined period of time; much of the available literature refers to gatherings exceeding 25,000 individuals [1]. Some MGs are spontaneous, whereas others will have been planned several years in advance, and include events as varied as royal weddings, the Olympic Games, or the Muslim Hajj pilgrimage [1].

A range of respiratory and waterborne diseases outbreaks have been reported at previous MGs, and are responsible for an estimated 14 out of 21 documented events, with occasional onward dissemination beyond the initial location of the MG [2]. A comprehensive review of infectious diseases at MGs highlights that influenza is the acute upper respiratory tract pathogen most commonly reported in these settings, partly because of its short incubation period and ubiquitous nature [2-4]. Influenza outbreaks have been reported in settings of varying scale, ranging from outbreaks in close living conditions (including troop ships [5] and airplanes [6,7]) to outbreaks in large public gatherings (such as the Winter Olympics in 2002 in Salt Lake City, USA[8] and the World Youth Day in July 2008 in Sydney [9]). Further, respiratory illness was the most common diagnosis made at a surveillance clinic during the 2008 Olympic and Paralympic Games held in Beijing and other cities of China [10]. More recently, increased 2009 pandemic influenza virus activity has been reported during several music festivals [11], and the pandemic transmission risk at the 2009 Hajj pilgrimage and the Asian South Games was deemed sufficiently important to prompt a strengthening disease surveillance systems and implementation of vaccination programs [2,12]. In addition, outbreaks of vaccine-preventable diseases can occur during MGs; for instance, measles outbreaks were reported during the 2008 European Football Championship in Austria and Switzerland, with onward transmission to France, Germany, and Spain [13]. Similarly, recurrent outbreaks of meningococcal meningitis have been well publicized during past Hajj pilgrimages, which attract millions of pilgrims, prompting mandatory vaccination of all participating visitors by the Government of Saudi Arabia.

Olympic events are perhaps the largest and most anticipated of all MGs, and yet, despite their scale, experience suggests that the probability of a large-scale infectious disease event is typically low. Indeed, the proportion of healthcare visits in Sydney during the 2000 Olympics for infectious diseases was less than 1%. In the 2006 winter Olympics in Torino, Italy, surveillance for acute gastroenteritis, influenza-like illnesses, measles, and other infections found incidences similar to non-Olympics time periods [14]. Similar experiences were reported in the earlier Summer Olympics in Atlanta (1996) and Los Angeles (1984) [10]. However, epidemiological alerts can happen, as shown by an anthrax alert at the Salt Lake City airport on the first night of the 2002 Winter Olympics, which was found to be due to an environmental sample that falsely tested positive [15].

The relatively low frequency of outbreaks reported during MGs could stem from several factors. MG events are naturally ideal settings for infectious-disease transmission because of the large numbers of dense contacts. However, the probability of observing a large-scale outbreak given the introduction of a particular infectious disease introduction is relatively small, owing to the high disease-extinction rates associated with high stochasticity in heterogeneous populations typical of MGs (for example, heterogeneity in background susceptibility, infectiousness, and vaccination rates) [16]. In addition, limited or overwhelmed disease surveillance systems can complicate early outbreak detection, reporting, and control during MGs. Moreover, for infectious diseases with long incubation periods such as tuberculosis, transmission may not be noticed during the time course of an MG event [2]. Overall, although the probability of large-scale epidemics arising from MGs seems, based on previous experience, to be low, such events do have the potential to generate unprecedented rates of morbidity and mortality at local and global levels (that is, these are low probability, high-impact events, similar to the risk of emergence of a novel pandemic virus).

A review of key data and methodological needs is useful to improve assessment of epidemic risk during MGs and to guide public-health interventions. In this article, we discuss modeling aspects and data requirements relating to modeling respiratory-disease outbreaks and responses during MG, with a specific focus on influenza and other respiratory diseases. The focus on respiratory diseases, particularly influenza, is based on the observations that respiratory infections are the most commonly reported diseases at MGs, and that influenza has been the subject of a rich modeling literature that can be used as a model for other pathogens. In particular, the A/H5N1 avian influenza threat and the 2009 A/H1N1 pandemic have helped improved influenza models and forecasts [17-22]. Because disease transmission during MG events is often tightly connected to the community at large via local and global transportation networks, we also considered onward transmission at broader spatial scales (city, region, world) and the relevant public-health control interventions. In the first section of this paper, we review key modeling concepts, and characterize the population and social network of MG participants. In the second section, we discuss how to incorporate these specificities into existing disease-transmission models. In the third section, we highlight key data gaps related to models of respiratory diseases at MG, and suggest innovative approaches to fill those gaps and better inform future models.

### Key disease-model concepts

The risk of infectious-disease transmission during MGs is directly related to the characteristics of the participants and their environment [2]. The effects of these factors on the risk of disease transmission can be integrated in key epidemiological quantities: the basic reproduction number, *R*_0_, and the effective reproduction number, *R *[23-25]. *R*_0 _measures the average number of secondary cases generated by a primary infectious individual in a completely susceptible population. A more practical quantity is the effective reproduction number, *R*, which quantifies the potential for infectious-disease transmission in a population that may be only partially susceptible owing to prior exposure or vaccination [26]. From a probabilistic perspective, *R *and *R*_0 _denote the mean of the distribution of secondary cases for each single primary case in the population to account for individual-level variation in, for instance, infectiousness and contact rates [16]. *R *can be formulated as the product of three quantities: the contact rate, the conditional probability of transmission per contact, and the duration of the infectious period [23,24], hence we can expect higher values for *R *in crowded or confined conditions associated with MGs. Overall, *R *of less than 1 indicates that a major epidemic is likely to occur whereas *R *of greater than 1 indicates that transmission chains cannot be sustained. Respiratory infections cover a wide range of transmission potentials, with *R *being estimated at 1.2 to 1.6 for seasonal influenza [27], 1.4 to 5.2 for pandemics [28-32], 15 for pertussis, 17 for measles [23], and 1.2 to 1.3 for meningococcal meningitis [33].

Another key quantity for disease control is the serial interval, which measures the time interval between successive cases and sets the time scale for epidemic growth, and hence the speed with which intervention measures need to be initiated [34]. Despite the relatively low transmission potential of influenza, outbreaks are difficult to control in real time because of its short serial interval of 2 to 3 days and the fact that a substantial fraction of transmission events occur before a case becomes symptomatic [34].

### Characterizing population at risk and contact networks during mass gatherings

#### Structure of disease-relevant contact network

Perhaps the most challenging aspect of modeling infectious-disease transmission in the context of MG lies in appropriately capturing the complexity of dynamic human interactions and contact networks to provide valid and reliable predictions of transmission potential and attack rates. The dynamic social contact networks during MGs will depend on a number of factors, including the type (for example, confined versus open setting), size and duration of the event, the schedule of activities, the capacity of the corresponding locations in which the activities take place (for example, Olympic stadium, aquatic center), and specific crowd behavior (for example, in the case of diseases spread by aerosols, the use and sharing of plastic blowing horns by sports fans to provide audible support for their team [35]).

Most contact-network surveys have been based on cumbersome questionnaires with arbitrary physical definitions of 'social' contacts between individuals, but recent technological advances in wearable sensing devices allows unobtrusive and unsupervised quantification of contact intensity and duration. Radiofrequency identification devices were recently used to monitor in great detail the face-to-face contact patterns relevant to the spread of infectious diseases [36], particularly in primary schools [37,38]. Such detailed analysis of contact patterns highlighted important departures from homogeneous mixing assumptions, which should be integrated in disease models focused on outbreaks in schools or on childhood infections [38-41]. Another recent study analyzed real-time close contact interactions between conference participants, and found that the duration of contacts between the participants provided a good approximation to the epidemic dynamics compared with results obtained by modeling the full dynamic contact network [42,43]. To our knowledge, no detailed survey of contacts has been performed at MG events. A key avenue for future research would be to gain more information on contact networks in this context, perhaps by distributing sensing devices to a sample of MG participants. Although it could be challenging to enroll representative populations of MGs, recent efforts have achieved 30% participation rates for conference settings [42]. Crowd modeling is another interesting research area that offers useful tools for modeling pedestrian flow and crowd dynamics at the individual level, particularly during MGs [44]. Of note, electronic devices such as mobile phones have improved the estimation of the sizes of crowds compared with capture-recapture methods [45].

#### Demographic characteristics

The demographic characteristics and particularly the age distribution of the MG participants could inform the parameterization of age-specific contact rates and pre-existing immunity [46,47], and thus the risk of severe disease outcomes [48,49]. For instance, school-age children tend to have high contact rates, are more susceptible to influenza infection, and have increased viral shedding relative to other age groups [50]. By contrast, populations of seniors experience relatively low influenza attack rates, but they are at higher risk of severe disease outcomes during seasonal influenza epidemics [51]. During pandemic seasons, however, senior populations may benefit from significant residual immunity to infection [52-54]. During the 2008 Olympic and Paralympic Games in Beijing, most (76%) of the patients at a surveillance clinic were between the ages of 16 and 54 years [10], suggesting a relatively low susceptibility of older MG populations to influenza infection and severe outcomes, relative to other age groups. Finally, rates of hand hygiene and disease reporting are likely to be reduced during MGs, but relevant data on this behavioral aspect is lacking.

#### Susceptibility levels and vaccination status

Susceptibility of the MG population to the occurrence of outbreaks is a function of the collective vaccination status, previous disease exposure history, and resulting immunity. It is important to take into account the country of origin of participants, as populations from different geographical areas are exposed to different pathogens. In addition, exposure and co-infections with multiple pathogens in populations from low-income and middle-income countries could also affect immune status to common infections, such as influenza [55]. Hence, it is important to know the expected composition of participants based on country of origin and expected immunization status in order to assess susceptibility of the total MG population, which could significantly differ from that of the local population. During the 2008 Olympic and Paralympic Games in Beijing, the foreign visitors came from 46 countries, but the great majority arrived from high-income temperate regions including the USA (24%), the Netherlands (19%), Australia (9%), and the UK (9%) [10]. In addition, only 9% of foreign visitors at a surveillance clinic reported having received the influenza vaccine in their country of origin [10].

#### Timing of MG in relation to travel patterns, climatic conditions, and school cycles

The risk of influenza transmission at MG is connected to incoming travel patterns, local climatic conditions, and school cycles. In particular, the transmissibility of influenza has been shown to be associated with environmental conditions, as low absolute humidity has been shown to favor virus transmission and survival in the laboratory [56-58]. Influenza has marked winter seasonal patterns in temperate areas of the world, with viruses being reintroduced every winter and causing large and intense outbreaks, followed by fade-out periods in warmer months, during which little influenza activity is detected [59]. By contrast, in the Tropics, the seasonality of influenza is less defined, and the timing of virus activity varies between locales [60,61]. As an example, the influenza outbreaks identified during the World Youth Day occurred during the regular influenza season in Australia in winter 2008 [62]. Similarly, schools have been associated with increased rates of influenza transmission at the community level [50], and the celebration of MGs during school activity periods could significantly increase the risk of epidemic events.

### Modeling infectious-disease transmission during mass gatherings

#### Stochastic versus deterministic models

**Figure 1:**
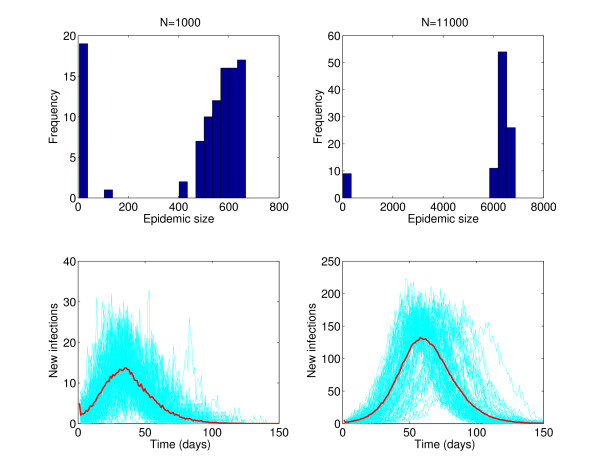
**Effect of demographic stochasticity on influenza epidemics**. The classic stochastic SEIR (susceptible, exposed, infectious, recovered) model tailored to the epidemiology of influenza, based on a latent period of 1.5 days, an infectious period of 3 days, basic reproduction number (*R*_0_) of 1.5, and an assumption of homogenous mixing of the population, was used to generate 100 stochastic simulations in two population sizes of n = 1,000 (left panels) and 11,000 individuals (right panels). Simulations were initialized with five infectious individuals. Histograms show a higher probability of epidemic extinction in the lower population setting. Stochastic epidemic realizations are shown in light blue, while the red solid line curve corresponds to the average of the stochastic realizations that resulted in epidemics.

Mathematical models of infectious-disease transmission are typically expressed as deterministic dynamical systems that capture the average epidemic behavior and are often amenable to mathematical analysis [23,24,63,64]. By contrast, the models most appropriate for MGs should include probabilistic components to integrate stochasticity in the risk of infection, especially in the case of smaller populations, in which demographic stochasticity will affect the risk of outbreak emergence. We produced stochastic simulations of the classic SEIR (susceptible, exposed, infectious, recovered) transmission model tailored to the epidemiology of influenza [65] (Figure 1). It is also important to use probabilistic models to consider shorter temporal scales during MGs and compare these with community-level transmission. Moreover, stochastic models allow the estimation of the probability that introduction of initial case(s) will trigger a major epidemic, which is also significantly affected by host heterogeneity (for example, age, vaccination status) and mixing patterns (for example, confined space in airplane, large conference hall).

#### Mass gathering contact structure

In general, infectious-disease models using explicit contact-network approaches can be classified as agent-based, individual-based, and spatially structured models, according to the level of detail used to model disease transmission. Agent-based models are very flexible, and can incorporate heterogeneities in the interactions, behaviors, and susceptibility of individuals, which make them particularly suitable for the analysis of collective dynamics of complex systems [66]. Models relying on detailed information on individual-level activities have been used to model the spread of rapidly disseminating infectious diseases, including influenza, and to help assess intervention strategies [19,20,67-73]. A slightly cruder approach is provided by individual-based models, which are often based on static networks of individual interactions [74,75]. Spatially structured epidemic models consider subsets of the population categorized by geographic location, with interactions between these subpopulations based on human mobility patterns [76-78] (for example, governed by gravity laws, whereby larger population centers tend to interact with higher probability) and age-specific contact rates based on contact survey data [46].

**Figure 2:**
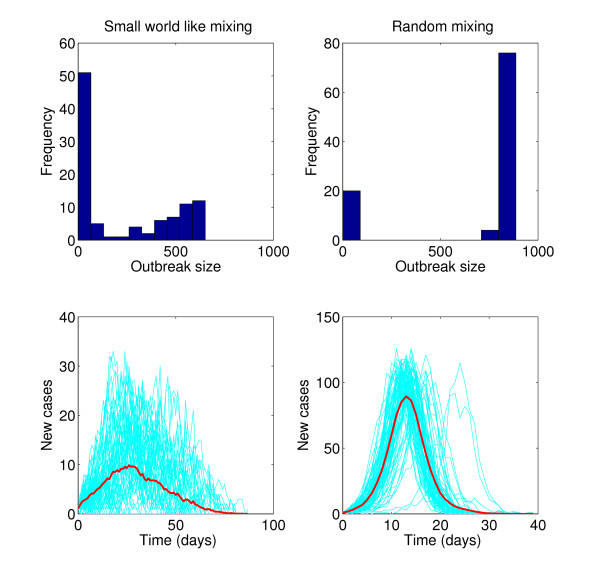
**Effect of contact-network structure on influenza epidemics**. The classic stochastic SEIR (susceptible, exposed, infectious, recovered) model was tailored to the epidemiology of influenza, based on a latent period of 1.5 days, an infectious period of 3 days, and fixed transmission probability per contact, simulated using two different contact networks: 1) a small-world contact network based on the model of Watts and Strogatz [74], with an average degree of 4 and disorder parameter (p) of 0.1 in a population of 1,000 individuals (left panels) and 2) in a random network model with an average degree of 4 (right panels) with the same population size. Histograms show the distribution of outbreak sizes for both network topologies when everything else is kept fixed. Epidemic realizations are shown in blue, while the red solid line curve corresponds to the average of the stochastic realizations that resulted in epidemics.

The choice of the underlying assumptions about the contact-structure profile will depend on the MG population. For instance, in the case of infectious-disease transmission in small populations within confined settings, such as disease transmission in Navy ships [79] and correctional facilities [64], assuming a well-mixed population could be reasonable. However, recent work has identified significant departures from homogenous mixing assumptions during specific MGs, including conference settings [42]. The role of the contact-network structure on transmission dynamics is illustrated in Figure 2; faster disease-transmission rates are seen in random-mixing structures than in small-world network structures [74].

#### Airborne transmission in confined spaces

Quantitative microbial risk-assessment models have been successful in modeling airborne transmission of respiratory pathogens in confined spaces, such as influenza and tuberculosis on airplanes [6,80-82], and quantifying infection risk as a function of host, pathogen, and environmental factors [83-86]. In particular, the Wells-Riley model [87-90] has been shown to be useful for well-mixed confined spaces, which can readily incorporate environmental and pathogen-specific variables such as room ventilation rates, pulmonary respiratory rates, number of infectious individuals, and infectivity, as a function of virus concentration exhaled by infectious individuals [91]. This model has been widely used in the microbial dose-response modeling literature [92], and was successful in assessing the risk of transmission in different areas of a closed-space environment and calculating the potential number of new infections generated over a specific time period.

Table 1 summarizes the differences between the quantitative risk-assessment and the dynamic transmission models. Dose-response models are traditionally used to understand dose-response mechanisms and allowable microbial concentration in foods and water, but they can also be combined with other epidemiological models. A typical example is a model for point source outbreaks caused by certain exposure doses (for example, the Sverdlovsk anthrax leak), which can provide estimates for the total number of cases, the time of exposure, and the dose [92,93]. Another useful approach is to account for the dose-response nature of the risk of infection in traditional disease-transmission models, allowing adjustment for different transmission probabilities by route of transmission (aerosols, droplets) [94].

**Table 1:** Contrasting quantitative microbial risk-assessment models and infectious disease-transmission models.

Modeling aspects	Quantitative microbial risk-assessment model	Dynamic transmission model
Non-linear dynamics	Usually no	Usually yes

Environmental sources	Yes	Usually no

Inclusion of uncertainty/stochasticity	Yes	Case-by-case basis (deterministic dynamical systems, stochastic, hybrid models)

Time scale	Days	Weeks to months

Population size	Thousands (music festival) to millions (Hajj pilgrimage)	Hundred thousands to millions

Population density	High	Low to high

Model structure	Spatial-temporal network (Summer Olympics); confined space (Army barracks)	Age-structured, random-mixing populations; patch models; household-level models; large-scale individual-level models

Stochastic disease extinction	Yes	Unlikely

Endemicity	No	Yes

Contribution of super-spreading events	High	Low to moderate

#### The role of multiple initial infectious sources

**Figure 3:**
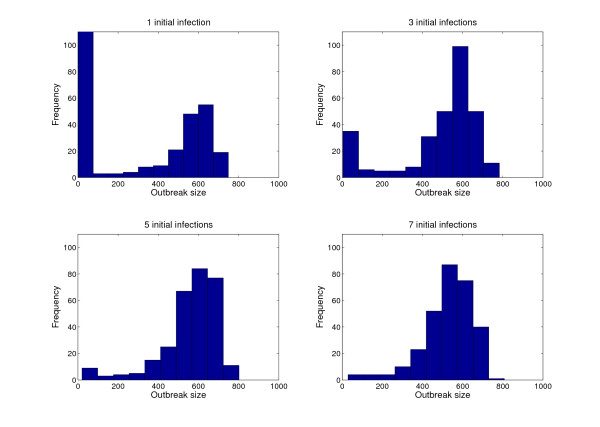
**Effect of the initial number of infectious individuals**. The classic stochastic SEIR (susceptible, exposed, infectious, recovered) model tailored to the epidemiology of influenza, based on a latent period of 1.5 days, an infectious period of 3 days, and fixed probability of transmission per contact, was simulated on small-world contact networks based on the Watts and Strogatz network model [74] with an average degree of 4 and disorder parameter (p) of 0.1 in a populations of 1,000 individuals. Initially infectious individuals were selected uniformly at random from the population. Histograms show how the distribution of outbreak sizes shifts to larger epidemics as the initial number of infectious individuals increases.

An important modeling consideration is the initial number of infectious individuals, and their geographical location and contact networks, particularly in the context of highly heterogeneous MG populations. Indeed, the probability of an epidemic unfolding and the rate of growth rate in the number of infections will depend upon the number of initial infectious individuals and their spatial location within the MG contact network [65]. This also has relevance to the potential deliberate release of infectious diseases during MGs. To illustrate this point, we show how outbreak size increases with the initial number of infectious individuals, using a small-world contact-network structure of 1,000 individuals (Figure 3).

#### Spatial scale considerations and global transmission models

Disease transmission during MG events cannot be disconnected from the rest of the population, because of the tight connections to the community at large via local, regional, and global transportation networks. For instance, in the context of large MGs such as the Olympics Games or the Soccer World Cups, given the scale of the event and the many neighborhoods and sometimes cities involved, models appropriate for a large city or a network of cities may be more appropriate than models limited to a confined space. Hence, large-scale transmission models and population-wide public-health control interventions are also useful to discuss in the context of MG events.

In the event of an infectious-disease outbreak during a MG, worldwide travel patterns originating from the MG population could disseminate the outbreak on a global scale within a matter of weeks [95]. For instance, following the identification of the 2009 A/H1N1 influenza pandemic in Mexico and California in late March 2009, the novel pandemic virus was detected within a few weeks in the 20 countries with highest volume of passengers arriving from Mexico [96].

Large-scale computational transmission models parameterized with high-volume air-traffic data and country-level seasonality factors are being increasingly used to assess the global transmission patterns of emerging infectious diseases and the effectiveness of control measures [18,77,97,98]. Such large-scale modeling efforts predicted that an early peak of pandemic influenza A/H1N1 virus activity would occur in October/November 2009 in the Northern hemisphere, several weeks before vaccination campaigns could be carried out, and that antiviral use could delay peak pandemic timing. In regards to the effects of interventions on global transmission patterns, multiple studies have concluded that substantial reductions in air travel could only provide a short delay in pandemic progression [99-102].

Overall, network-based approaches have become useful tools to model the transmission dynamics of infectious diseases and control interventions on city, regional, and global scales [19,20,67-69,74,103,104]. These simulation models are currently underused in the context of MGs. To our knowledge, only a single network-based modeling study has examined how outbreak size may depend on the timing of an MG event, relative to the temporal course of an influenza pandemic [105]. The MG event was modeled as a change in population mixing.

### Key data needs for large-scale simulation models

Global transmission models rely on large amounts of data on demographics, population movements, and age-specific contact rates. High-resolution demographic and age-specific contact data has become available for a number of areas, including the USA [19,106], and southeast Asia [20,107], while age-specific contact rates have been derived from population surveys for a number of European countries [46]. However, travel patterns before and after the MG event are naturally difficult to ascertain well in advance of the event, but are essential data for anticipating the expected composition of the MG and the potential global infectious disease-transmission patterns. Analysis of international travel data from previous Summer Olympics suggests that changes in travel patterns to the host city are difficult to predict months in advance [12]. Moreover, high-resolution contact rate data and within-country connectivity data are not available for most countries. Equally important is the need for country-specific historical immunization coverage data to assess the risk of importation to and from different locations. Finally, additional information on the temperature, humidity, ventilation settings, and capacity of confined environments specific to MGs, such as indoor stadiums (Olympics) and mosques (the Hajj), would be useful to tailor environmental risk models to MGs.

### Public-health interventions during mass gatherings

The decision-making process on the type and intensity of interventions to put in place to control a MG outbreak will depend on our ability to detect early cases, characterize the transmission potential and severity of the associated pathogen, and implement interventions rapidly [108]. Overall, the timing of start of interventions will depend on the epidemiology of the infectious disease and the availability of surveillance data that is representative of the general population. Cancellation of large public gatherings has been successfully implemented during past influenza pandemics [4,9,25,109]; however, cancellation of a major MG event such as the Summer Olympics as a result of perceived infectious-disease transmission risk could be counterproductive, because of potential for rapid global spread. Instead, recommendations to use face-masks and increase hygiene measures could prove to be effective mitigation strategies, together with preventive or reactive vaccination in the early stages of the outbreak, as shown in past epidemics of influenza, meningococcal meningitis, and measles [69,110-114]. This is particularly important in light of outbreaks of measles and mumps reported at past MGs [2], and the difficulty in reaching the critical vaccination coverage rate against childhood infectious diseases in many European countries [115]. The importance of putting in place targeted vaccination strategies against influenza and other respiratory pathogens prior to any MG event cannot be overemphasized.

In the extreme situation in which evidence suggests the potential for increased rates of hospitalization and mortality, stringent interventions could be justifiable, including imposing movement restrictions on MG participants to avoid or slow the importation of cases [86] to high-risk countries, concurrent with a reactive vaccination strategy [116].

The composition and dynamics of MG events as large as the Olympics are complex. Recent data indicate that influxes of several million visitors are typically expected, including tens of thousands of journalists and athletes from a couple of hundred nationalities. Moreover, competitions take place in a number of open and confined settings, with capacities ranging from a few thousand to tens of thousands. The expected age distribution of participants is relatively young, with the majority being young and middle-aged adults [10], which is the age group that experienced the highest death rates during the most recent influenza pandemic [117]. Moreover, recent data indicate that only a small fraction of the participant population is expected to have received the seasonal influenza vaccine in their country of origin before the start of the competitions [10,118]. Public-health preparedness planning is very intense during Olympic Games. This was illustrated by the 2012 London Summer Olympics, for which the UK Health Protection Agency set up an enhanced disease-monitoring system including laboratory surveillance, clinical case reporting, and syndromic surveillance based on patient symptoms [119-121], and surveillance was conducted using a lower than usual detection threshold. However, to our knowledge, infectious disease-transmission models were not integrated in any of these preparedness efforts.

### Future directions

Below we summarize the data and modeling gaps that we have identified throughout this review, which must be filled to improve infectious-disease models for MGs. Because no detailed survey of MG contact networks has been carried out, a key avenue for future research would be to gain more information on the patterns and duration of human interactions during MG events, perhaps by distributing innovative contact-sensing devices to a sample of MG participants [36]. The Olympic Games, Soccer World Cups, and annual Hajj pilgrimages offer an interesting opportunity to study large crowds in the context of infectious-disease transmission. Although it is not feasible to monitor contact patterns in the entire MG population, information from a representative sample would be useful, and participation in previous contact-sensing device studies has been high. Ideally, such studies should be combined with enhanced monitoring of disease activity, including simultaneously testing for several pathogens (for example, using multiplex PCR) and incorporating innovative approaches for disease surveillance (for example, exploiting web-based technologies, and data-gathering and dissemination methods via smart phones) [122].

Comprehensive modeling studies are lacking to assess the cost-effectiveness of intervention strategies such as movement restrictions and reactive vaccination in the context of infectious-disease transmission during MGs. This is partly because of our limited knowledge of crucial demographic and susceptibility characteristics of the population and the relevant contact structure for disease transmission. In parallel, the higher levels of computational power now available is facilitating the development of extremely detailed transmission models at multiple spatial scales ([18,19,22,107,123]. Another key data gap is the difficulty in ascertaining up-to-date air-travel patterns relating to MG events as well as country-specific repositories of demographic, contact rates, and immunization data for childhood and other infectious diseases, which would be needed for appropriate calibration of large-scale transmission models involving large numbers of international visitors. Finally, it would be useful to validate infectious-disease models for MGs against historical outbreaks that have been well documented in the literature, especially given the stochastic nature of these outbreaks in heterogeneous populations [2].

We note that the number of efforts to integrate microbial risk-assessment modeling and dynamic population-level transmission modeling approaches remains limited [92-94]. Hence, the integration of quantitative risk models into large-scale dynamic transmission models has the potential to improve predictive capabilities in relation to epidemic transmission patterns and prospects for outbreak control, particularly in the context of disease transmission at MGs. Such modeling approaches could follow a hierarchical structure by connecting disease-transmission processes on different spatial scales [86].

## Conclusions

Comprehensive modeling studies are needed to assess the cost-effectiveness of intervention strategies against infectious disease arising during large MGs such as the Summer Olympics and the annual Hajj events. These studies will heavily rely on our ability to quantify population mixing characteristics during MG events; anticipate air-travel patterns before and after the MG event; gather country-specific repositories of demographic factors, contact rates, and immunization rates for childhood and other infectious diseases; and estimate the potential effect of collective behavioral changes during MG events. Further, development of novel mathematical and statistical approaches specific to MGs, and integration of existing approaches, would be useful to provide more appropriate models, which could be tested against historical events [124]. Finally, MG preparedness and contingency intervention plans to mitigate infectious-disease transmission could incorporate some of these modeling research, inspired by influenza modeling efforts that have helped shape pandemic preparedness plans in recent years [18,19,22,99-102,107,123].

## Competing interests

The authors declare that they have no competing interests.

## Authors' contributions

All the authors contributed to the writing and editing of the manuscript. All the authors have read and approved the manuscript for publication.

## Pre-publication history

The pre-publication history for this paper can be accessed here:

http://www.biomedcentral.com/1741-7015/10/159/prepub
